# Unilateral Clearance for Primary Hyperparathyroidism in Selected Patients with Multiple Endocrine Neoplasia Type 1

**DOI:** 10.1007/s00268-016-3624-9

**Published:** 2016-07-11

**Authors:** Wouter P. Kluijfhout, Toni Beninato, Frederick Thurston Drake, Menno R. Vriens, Jessica Gosnell, Wen T. Shen, Insoo Suh, Chienying Liu, Quan-Yang Duh

**Affiliations:** 1 Department of Surgery, University of California, Mt Zion, 1600 Divisadero Street, San Francisco, CA 94115 USA; 2 Department of Surgery, University Medical Center Utrecht, Utrecht, The Netherlands; 3 Division of Endocrinology, University of California, San Francisco, CA USA

## Abstract

**Background:**

Primary hyperparathyroidism is the most common manifestation of multiple endocrine neoplasia type 1 (MEN1). Guidelines advocate subtotal parathyroidectomy (STP) or total parathyroidectomy with autotransplantation due to high prevalence of multiglandular disease; however, both are associated with a significant risk of permanent hypoparathyroidism. More accurate imaging and use of intraoperative PTH levels may allow a less extensive initial parathyroidectomy (unilateral clearance, removing both parathyroids with cervical thymectomy) in selected MEN1 patients with primary hyperparathyroidism.

**Methods:**

We performed a retrospective cohort study at a high-volume tertiary medical center including patients with MEN1 and primary hyperparathyroidism, who underwent STP or unilateral clearance as their initial surgery from 1995 to 2015. Unilateral clearance was offered to patients who had concordant sestamibi and ultrasound showing a single enlarged parathyroid gland. For both the groups, we compared rates of persistent/recurrent disease and permanent hypoparathyroidism.

**Results:**

Eight patients had unilateral clearance and 16 had STP. Subtotal parathyroidectomy patients were younger (37 vs 52 years). One patient in each group had persistent disease. One (13 %) unilateral clearance and five (31 %) STP patients had recurrent hyperparathyroidism after a mean follow-up of 47 and 68 months (*p* = 0.62). No unilateral clearance patients and two of 16 SPT patients had permanent hypoparathyroidism (*p* = 0.54).

**Conclusions:**

Some MEN1 patients with primary hyperparathyroidism who have concordant localizing studies may be selected for unilateral clearance as an alternative to STP. For appropriately selected MEN1 patients, unilateral clearance can achieve similar results as STP and has no risk of permanent hypoparathyroidism, and may facilitate possible future reoperations.

## Introduction

Primary hyperparathyroidism is often the first manifestation of multiple endocrine neoplasia type 1 (MEN1) with 100 % penetrance by age 65 [1]. Patients with MEN 1 usually have multiple abnormally appearing parathyroid glands (“asymmetrical hyperplasia”). In contrast, 80 % of patients with sporadic primary hyperparathyroidism have a single adenoma [2]. Since all the glands in patients with MEN1 have the *menin* mutation, any parathyroid tissue left in the patient is at risk for growing and developing into a hyperfunctioning gland. To achieve the lowest possible rate of recurrent disease, surgeons traditionally have advocated most aggressive resection leaving as little parathyroid tissue as possible [3, 4]. Therefore, current guidelines recommend performing either subtotal (3 1/2) parathyroidectomy or total parathyroidectomy with forearm autotransplantation at initial operation [5]. Aggressive resection of parathyroid glands, however, also leads to a higher risk of permanent hypoparathyroidism, which can greatly affect patients’ quality of life [3, 6–10].

Several reports have shown that some patients do not develop recurrent disease even after long follow-up, despite less aggressive initial operation [8, 11]. With the increasing ability to preoperatively localize large and hyperfunctioning parathyroid tumors and intraoperatively monitor PTH levels, some surgeons have begun to perform less aggressive initial parathyroidectomy in order to lower the risk of permanent hypoparathyroidism [8, 9]. To avoid re-exploration in a scarred neck, a new strategy has been proposed. During a “unilateral clearance,” both the abnormal gland and other parathyroid tissues from the same side of the neck (the other parathyroid and the cervical thymus) are removed in patients with MEN1 and primary hyperparathyroidism. We retrospectively reviewed the results of unilateral clearance in eligible MEN1 patients in our institution over the past 15 years. We examined the rates of permanent hypoparathyroidism and persistent or recurrent disease in this cohort and compared them to the results of traditional STP.

## Material and methods

### Surgical strategy for primary hyperparathyroidism in MEN1 patients

Our previous standard surgical approach to MEN1 patients with primary hyperparathyroidism was STP. Preoperative localization studies were not routinely performed. A traditional bilateral neck exploration was performed, with inspection of all four parathyroid glands and identification of potential supernumerary glands. The most normal-appearing gland was then biopsied, marked with a clip, and a 50-mg remnant was left in situ to prevent postoperative hypoparathyroidism. The other three glands as well as the cervical thymus were removed [11].

Since 2000, with increasing and routine use of preoperative sestamibi scans and ultrasound, we began to identify a subgroup of MEN1 patients with a preoperatively located single abnormal parathyroid gland. For this subgroup of patients, we start the operation by finding and excising the abnormal gland, followed by excising the other gland on the same side as well as the ipsilateral cervical thymus. We use intraoperative PTH (IOPTH) monitoring to look for a significant drop. If the 10-min postexcision PTH adequately drops by more than 50 % from baseline, the other side is not explored.

### Patients

All patients with MEN1 and primary hyperparathyroidism, who had their initial operation at UCSF between 1995 and 2015 were included in the study. The diagnosis of MEN1 was based on (1) proven genetic mutation, (2) coexisting endocrine neoplastic disease in the pancreas, duodenum or pituitary gland, or (3) at least one first-degree relative with MEN1 [5]. This study was reviewed and approved by the local institutional review boards.

### Outcomes

Persistent hyperparathyroidism was defined as elevated serum calcium levels with inappropriately elevated levels of PTH within 6 months of initial parathyroidectomy, and recurrent hyperparathyroidism as increased levels of calcium and PTH after at least 6 months of follow-up with normalization of serum levels of calcium and PTH after initial parathyroidectomy. Permanent hypoparathyroidism was defined by serum calcium levels below the normal range and requirement for supplemental calcium and vitamin D after 6 months following operation.

Serum calcium was measured using a UniCel DxC 800 Clinical system (Beckman Coulter), with reference level 8.8–10.3 mg/dL. Intraoperative PTH was measured using a UniCel DxI 600 Immunoassay system (Beckman Coulter). Intact PTH was measured with Immulite 2000 Immunoassay (Siemens Healthcare), with reference level of 12–65 ng/L.

### Statistical analysis

Standard descriptive statistics (mean, range and frequency) were used to analyze patients and disease characteristics. Fisher’s exact test was used to compare incidence of recurrent disease, persistent disease, and permanent hypoparathyroidism. Student *T*-test was used to compare continuous variables between the two surgical groups. A *p* *v*alue of less than 0.05 was considered statistically significant. All data analyses were performed using SPSS version 21.0 (SPSS Inc., Chicago, IL).

## Results

Eight patients underwent unilateral clearance during the study period. There were five (63 %) women and three men; the mean age was 52 (Table 1). After a mean follow-up of 47 (range 2–112) months, one patient (Patient 2) developed recurrent disease 8 years after initial operation, with a CT scan showing a lesion suspicious for parathyroid adenoma in the left tracheoesophageal groove on the same side as the initial unilateral clearance, and is being treated with cinacalcet due to the interval development of widely metastatic neuroendocrine tumor. Patient 8 had persistent disease. She underwent a right-sided unilateral clearance of the abnormal lower gland shown by concordant sestamibi and ultrasound and the upper ipsilateral gland and cervical thymus, with a drop in IOPTH from 389 ng/L preincision to 109 ng/L postexcision (via internal jugular). 2 weeks postoperatively, she had elevated serum levels of PTH (94 vs 138 ng/L preoperatively) and calcium (10.5 vs 11 mg/dL preoperatively). Repeat imaging with US and sestamibi showed an additional abnormal left lower parathyroid. She then underwent exploration of the left side, with excision of a 1.5-cm tumor and biopsy of the other gland. After this second operation, she had transient hypocalcemia, but has remained normocalcemic since then (50 months of follow-up). No patient had permanent hypoparathyroidism.

**Table 1:** Multiple endocrine neoplasia type 1 patients (*n* = 8) who underwent unilateral clearance for primary hyperparathyroidism

Patient	Sex	Age	Other MEN1 tumors	Preoperative		Persistent disease	Recurrent disease	Hypoparathyr-oidism	Follow-up months
				Calcium	PTH				
1	F	46	Pituitary	13.2	583	No	No	No	2
2	M	33	Gastrinoma	10.2	106	No	Yes^a^	No	106
3	F	35	Insulinoma	11.1	127	No	No	No	112
4	M	65	Pituitary	9.8	204	No	No	No	33
5	F	68	Pituitary Pancreatic	10.9	90	No	No	No	10
6	M	65	Pituitary	11.5	92	No	No	No	6
7	F	51	Pancreatic	11.4	115	No	No	No	59
8	M	59	−^b^	11.2	138	Yes	No	Temporary^c^	50

*F* female, *M* male, *PTH* parathormone

^a^Eight years after initial parathyroidectomy

^b^Diagnosis based on genetic mutation

^c^After reoperation

The 16 MEN1 patients who underwent STP during the same period were significantly younger than those who underwent unilateral clearance (37 vs 52 years, *p* = 0.027) (Table 2). None of these patients were scheduled for initial unilateral clearance, since they either did not have concordant imaging for one abnormal gland, or due to surgeon preference. After a mean follow-up of 68 (range 1–223) months, five of 16 patients had recurrent disease at 2, 5, 8, 8, and 15 years, respectively. One patient underwent a right neck re-exploration with removal of the residual right lower gland with normocalcemia at follow-up. One patient underwent an unknown second operation at an outside center, which did not cure her hyperparathyroidism, and is currently being treated with cinacalcet. One patient had a successful reoperation (left neck re-exploration with removal of regrown left lower parathyroid) but again recurred 3 years after the second operation and is now being medically managed. One patient had a completion parathyroidectomy with failed autotransplant to the forearm and developed permanent hypoparathyroidism. The last patient did not undergo a second surgery due to comorbidities and is also being medically managed. One patient who had persistent disease after an STP was followed for 3 years and then underwent a successful resection of the remnant right upper parathyroid with autotransplant. Overall, two of the 16 patients developed permanent hypoparathyroidism, one after initial STP and one after reoperation.

**Table 2:** Comparison between patients who underwent subtotal parathyroidectomy and those who underwent unilateral clearance for primary hyperparathyroidism

	Subtotal parathyroidectomy *N* = 16	Unilateral approach *N* = 8	*P* value
Age, mean (range)	37	52	0.027
Female	12 (75 %)	5 (63 %)	0.647
Preoperative level of			
Calcium Parathormone	10.8	11.2	0.377
	112	182	0.147
Permanent hypoparathyroidism	2 (13 %)	0 (0 %)	0.536
Persistent disease	1 (6 %)	1 (13 %)	1.000
Recurrent disease	5 (31 %)	1 (13 %)	0.621
Follow-up (months)	68	47	0.454

## Discussion

In this study, we found that unilateral clearance can be performed in a subgroup of MEN1 patients with primary hyperparathyroidism who have concordant preoperative sestamibi and ultrasound localization studies. In these selected patients, using this less aggressive approach, the risk of permanent hypocalcemia is lower without a significantly increased risk of recurrent or persistent disease.

There are three goals for parathyroidectomy in MEN1 patients: correct hypercalcemia, avoid permanent hypoparathyroidism, and facilitate potential future operation [12]. The current MEN1 guidelines focus primarily on correcting hypercalcemia, both immediately (avoid persistent disease) and in the long term (avoid recurrent disease). Most experienced parathyroid surgeons recommend either STP or total parathyroidectomy with autotransplantation [3, 5–13]. Both have low recurrence rates, but still have some risk of permanent hypoparathyroidism.

Conceptually, the current recommendations apply the same operation to all patients with MEN1 using a relatively aggressive operation in order to lower the risk of recurrence. It is very likely, however, that not all MEN1 patients have the same aggressive parathyroid disease. There are more than 1000 germline mutations reported [14–16]. If we hypothesize MEN1 parathyroid disease to have a spectrum of aggressiveness (defined in terms of number of parathyroid glands involved), then conceptually it is not wrong to treat those with less aggressive disease with a less aggressive parathyroid operation. Once we make this conceptual leap, then the question becomes how do we identify those MEN1 patients who may have a less aggressive manifestation of hyperparathyroidism and treat them less aggressively.

It is possible that genetic heterogeneity will eventually help us to predict the aggressiveness of parathyroid disease [14]. The first proposal for a subdivision based on genotype was published in 2000 by Bartsch et al [17]. In a study investigating the outcome of patients with pancreaticoduodenal tumors, they found that truncating nonsense or frameshift mutations in the N- or C-terminal regions (exons 2, 9, 10) had a significantly higher rate of malignant tumors. Using this same genotype-phenotype comparison for primary hyperparathyroidism, another study has shown that after surgical intervention with less than STP, patients with these nonsense or frameshift mutations in exons 2, 9, and 10 have a significantly lower risk of persistent or recurrent disease than patients with other mutations [14]. Unfortunately, exact genetic mutations were only available in 7/24 (29 %) of our cases, and therefore we could not perform such a subanalysis. For now, however, localizing studies may help us identify a subgroup of patients that can be treated with less aggressive surgery.

Our patients who underwent unilateral clearance are a highly selected group. These patients had two concordant localizing studies for a single tumor. Localizing studies such as sestamibi and ultrasound are only about 80–90 % accurate for single gland diseased and much less accurate for patients with multiple gland disease, and therefore cannot be relied on completely for surgical decision-making [18, 19]. However, even in MEN1 patients, these localization studies may distinguish those with multiple large tumors from those with a single tumor. In other words, the localizing studies are used not just to find tumors but also to categorize patients into those who are more or less likely to have multiple large tumors. The patients who have only a single radiographically identified tumor are a different group and are more likely to have less aggressive parathyroid disease. This concept is supported by the observation that patients undergoing unilateral clearance were significantly older at parathyroidectomy. It is therefore not that surprising that the recurrence rate in this group was not higher than those who underwent a more aggressive operation of STP. Of the patients with at least 6 months follow-up, recurrent disease occurred in only one (14 %) of the seven unilateral clearance patients versus five (38 %) out of 13 patients who underwent STP. Although the follow-up for the latter was substantially longer, 83 versus 53 months, respectively, this difference was not significant.

In the unilateral clearance group, one patient had persistent disease and underwent contralateral exploration within 2 weeks of the initial surgery. The initial ultrasound, however, should not have missed this second 1.5 cm large tumor which was clearly visible at follow-up ultrasound 2 weeks later. This finding underscores the fact that ultrasound is operator dependent. In addition, during the initial surgery, the PTH dropped from 389 preincision to 109 postexcision. Even though this is a >50 % drop, PTH was still substantially elevated and should have raised suspicion for additional unresected disease. In all other cases, IOPTH dropped into normal range (<65 ng/L). This finding supports the observation that not all patients with a drop of at least 50 % in PTH are cured and the absolute postoperative PTH level should be taken into account as well [20]. One patient had recurrent disease 8 years after unilateral clearance. The recurrence was on the same side of the initial operation. He would likely have had a similar recurrence, if he initially had an STP, since it was a supernumerary gland.

The second goal of MEN 1-related parathyroidectomy is the prevention of permanent hypoparathyroidism. The same studies that showed a favorable outcome in terms of persistent and recurrent disease for (sub) total parathyroidectomy versus less than STP revealed that between 7 and 67 % of patients suffer from permanent hypoparathyroidism, greatly affecting patients’ quality of life [3, 6–10]. In our study, none of the patients who underwent the unilateral approach developed permanent hypoparathyroidism versus two patients who underwent STP. Although the difference was not significant due to our small sample size, the result is supported by the literature showing less risk of hypoparathyroidism for patients who undergo less than STP [8, 9, 21, 22].

The last goal of surgery in these patients is the facilitation of future surgery. During the unilateral clearance, both ipsilateral glands as well as the thymus are removed, and the neck is carefully inspected for supernumerary glands. By using this strategy, we reduced the likelihood of re-exploring the same side of the neck in case of persistent or recurrent disease. As suggested by the guidelines, doing less than STP might indeed be associated with a higher incidence of recurrent disease at long-term follow-up. However, there is often an asynchronous presentation of parathyroid tumors, which thus resecting only the large hyperfunctioning glands may serve as an alternative operation with fewer complications [9]. As shown by our data, patients undergoing unilateral clearance can be normocalcemic for years, with no risk of permanent hypoparathyroidism associated with (sub) total parathyroidectomy.

Traditionally, reoperation is considered a failure, but in the case of MEN1-related primary hyperparathyroidism, we recommend redefining goals and expectations. Balancing potential risks and consequences of permanent hypoparathyroidism, an initially less aggressive resection appears to be appropriate in the subgroup of patients with concordant imaging showing a single tumor. If a second operation is necessary, it will most likely be in the previously nonoperative side of the neck.

There are several important limitations to this study. The group that underwent unilateral clearance was highly selected, and may be genetically different from the group that underwent STP. The results found can therefore potentially be explained by disease biology rather than the surgery that was performed. Furthermore, our limited sample size may not have been sufficient to detect differences between the two groups. A recent publication from the National Institute of Health regarding this topic included 99 patients with MEN1. Although they concluded that limited parathyroidectomy was a setup for failure, they had only eight patients with concordant imaging studies similar to our patients, and no subanalysis was performed on this group [4]. A multicenter study will be helpful to address this shortcoming. In addition, our limited follow-up period may underestimate the true incidence of recurrent disease.

Nevertheless, we believe that our results clearly show that there is a subgroup of MEN1 patients with concordant imaging studies who could benefit from unilateral clearance with resultant lower risk of permanent hypoparathyroidism. The potential need for subsequent reoperation should not be considered a surgical failure, but a calculated risk in order to maximize the opportunity to prevent complications at the initial surgery.
